# Indoxyl Sulfate Elevated Lnc-SLC15A1-1 Upregulating CXCL10/CXCL8 Expression in High-Glucose Endothelial Cells by Sponging MicroRNAs

**DOI:** 10.3390/toxins13120873

**Published:** 2021-12-07

**Authors:** Yu-Chin Huang, Tzu-Chun Tsai, Chia-Hsin Chang, Kuo-Ting Chang, Pin-Hao Ko, Liang-Chuan Lai

**Affiliations:** 1 Graduate Institute of Physiology, College of Medicine, National Taiwan University, Taipei 10051, Taiwan; huangkatiecelia@gmail.com (Y.-C.H.); q.esther.chang@gmail.com (C.-H.C.); 2 Division of Nephrology, Taoyuan General Hospital, Ministry of Health and Welfare, Taoyuan 33004, Taiwan; 3 Department of Pediatrics, Taoyuan General Hospital, Ministry of Health and Welfare, Taoyuan 33004, Taiwan; mmozart@mail2000.com.tw; 4 Department of Public Health & Medical Humanities, Faculty of Medicine, School of Medicine, National Yang Ming Chiao Tung University, Taipei 11221, Taiwan; 5 Translational Medicine Center, Taoyuan General Hospital, Ministry of Health and Welfare, Taoyuan 33004, Taiwan; kklkimonster@gmail.com; 6 Department of Traditional Chinese Medicine, Taoyuan General Hospital, Ministry of Health and Welfare, Taoyuan 33004, Taiwan; 7 Bioinformatics and Biostatistics Core, Center of Genomic and Precision Medicine, National Taiwan University, Taipei 10055, Taiwan

**Keywords:** long noncoding RNAs, human umbilical vein endothelial cells, indoxyl sulfate, chemokines, diabetes mellitus, diabetes kidney disease, cardiovascular disease

## Abstract

Cardiovascular disease (CVD) is the leading cause of mortality in diabetes mellitus (DM). Immunomodulatory dysfunction is a primary feature of DM, and the emergence of chronic kidney disease (CKD) in DM abruptly increases CVD mortality compared with DM alone. Endothelial injury and the accumulation of uremic toxins in the blood of DM/CKD patients are known mechanisms for the pathogenesis of CVD. However, the molecular factors that cause this disproportional increase in CVD in the DM/CKD population are still unknown. Since long non-protein-coding RNAs (lncRNAs) play an important role in regulating multiple cellular functions, we used human endothelial cells treated with high glucose to mimic DM and with the uremic toxin indoxyl sulfate (IS) to mimic the endothelial injury associated with CKD. Differentially expressed lncRNAs in these conditions were analyzed by RNA sequencing. We discovered that *lnc-SLC15A1-1* expression was significantly increased upon IS treatment in comparison with high glucose alone, and then cascaded the signal of chemokines *CXCL10* and *CXCL8* via sponging *miR-27b*, *miR-297*, and *miR-150b*. This novel pathway might be responsible for the endothelial inflammation implicated in augmenting CVD in DM/CKD and could be a therapeutic target with future clinical applications.

## 1. Introduction

Diabetes mellitus (DM) is a metabolic disease characterized by dysregulation of glucose. Previous studies indicated inextricable links between DM and cardiovascular disease (CVD), which is the leading cause of mortality in the DM population [1]. Accumulating evidence has indicated that the immunopathological and inflammatory cascades play an important role in the initiation and development of CVD [2]. Chronic inflammation and immune system dysfunction are characteristic of DM and can produce a plethora of cytokines and chemokines, thereby contributing to CVD events [3,4,5,6]. For example, IP-10, encoded by *CXCL10*, and IL-8, encoded by *CXCL8*, belong to the C-X-C chemokine subfamily [7], and the literature is overflowing with evidence regarding their potential roles in causing atherosclerosis [8,9,10].

Diabetic kidney disease (DKD), a consequence of DM with chronic kidney disease (CKD), abruptly increases CVD mortality rate compared with diabetes alone [11]. For CKD patients, one of the most important triggers of CVD is endothelium dysfunction. This happens via the accumulation of various uremic toxins (UTs) that have a deleterious impact on endothelial cells [12]. Among these UTs, the protein-bound solute, indoxyl sulfate (IS), has been shown to be associated with high cardiovascular risk and mortality [13]. IS is derived from dietary protein and is metabolized into indole in intestinal bacteria, and then excreted in urine [13]. In CKD patients, IS accumulates in the blood. Many studies have reported that IS is a nephrovascular toxin that increases the expression of inflammation-associated genes [14] and thereby contributes to high cardiovascular risk and mortality [15,16]. In the early stage of DKD, serum levels of IS revealed a negative correlation with renal function [17]. Additionally, IS levels were higher in the DM patients with significant CAD and associated with renal function deterioration, inflammation, arterial stiffness, and coronary atherosclerosis [18,19]. Indoxyl sulfate seemed to be one of the “missing links” between DKD and prevalence of CVD. However, the causal effect of IS and CVD in DKD is still uncertain. Although systemic inflammatory activity is an established pathogenic mechanism of CVD in the context of DM and/or CKD, the specific molecular factors contributing to the augmented incidence of CVD events in DKD remains unknown.

Compelling evidence has demonstrated that environmental factors affect divergent gene expression through epigenetic mechanisms, such as microRNA and long non-protein-coding RNA (lncRNA) expression, and ultimately trigger crucial pathophysiological pathways of DM and its complications, such as CVD [20,21]. Numerous studies have reported the microRNAs involved in every stage of CVD events [22]. However, the role of lncRNAs in CVD is an emerging area of research. lncRNAs, which have a size of >200 nucleotides, have been found to be associated with DM onset, progression, and complications, impacting all aspects of the endothelial function and relieving UT-induced cell injury [23,24,25,26,27,28,29,30,31,32]. Therefore, we hypothesized that lncRNAs could be involved in the pronounced augmentation of CVD mortality in DKD.

In this study, human umbilical vein endothelium cells (HUVECs) were used to investigate the factors associated with CVD mortality in DKD, as endothelium injuries are one of the most important pathogenic mechanisms in CVD. HUVECs were cultured in high glucose to mimic the background of DM and treated without/with IS to mimic the endothelial injuries seen in DKD. Differently expressed lncRNAs in these two groups were hypothesized to be involved in the augmentation of CVD in DKD. We discovered that *lnc-SLC15A1-1* expression was significantly increased by IS treatment, and then cascaded the signal of *CXCL10* and *CXCL8* via sponging miRNAs.

## 2. Results

### 2.1. Upregulation of Lnc-SLC15A1-1 in High-Glucose-Cultured HUVECs after IS Treatment

**Figure 1:**
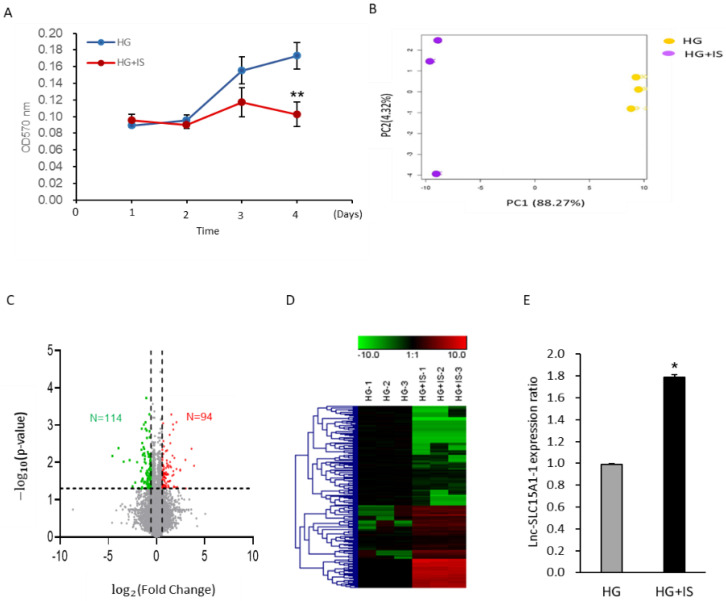
Upregulation of *lnc-SLC15A1-1* in the high-glucose-cultured HUVECs after indoxyl sulfate treatment. (**A**) MTT assay. Proliferation of HUVECs was examined in 30 mM D-glucose (HG) or HG with 0.1 mM indoxyl sulfate (HG + IS) for 4 days. (**B**) Principal component analysis (PCA) of samples. RNA sequencing was performed in HG + IS and HG groups (*n* = 3). Log-transformed fragments per kilobase of transcript per million mapped reads were used to perform PCA. (**C**) Volcano plot of differentially expressed lncRNAs. Selection criteria: fold change ≥1.5 or ≤−1.5 with *p*-value < 0.05. (**D**) Heatmap and hierarchical cluster analysis of 208 differentially expressed lncRNAs. Each column represents one sample, and each row represents one lncRNA. Red: upregulated lncRNAs; green: downregulated lncRNAs. (**E**) Relative expression levels of *lnc-SLC15A1-1* by quantitative RT-PCR. (* *p* < 0.05, ** *p* < 0.01).

To identify factors potentially involved in the augmentation of vascular events in DM patients, we treated HUVECs, representing endothelial cells, with HG and HG + IS. First, the proliferation of HUVECs growing in HG and HG + IS was examined using MTT assays. The growth rate declined significantly (*p* < 0.01) in the HG + IS group (Figure 1A). Subsequently, RNA sequencing was used to identify lncRNAs involved in this phenotype (GSE185598). In total, 72,915 transcripts were used in the latter analysis after removing those with zero value in all samples. As shown in Figure 1B, the expression profiling of total lncRNAs between these two groups was shown by principal component analysis (PCA). The data points for HG and HG + IS groups were clustered separately from each other, indicating the different lncRNA expression patterns between HG and HG + IS-treated cells.

By the selection criteria of FC (fold change) (≥1.5 or ≤−1.5 and *p*-value < 0.05), we identified 208 differentially expressed lncRNAs, 94 with upregulation and 114 with downregulation (Figure 1C). The expression profiling was illustrated in a heatmap with hierarchical clustering (Figure 1D). Two lncRNAs that passed primer design test from the top 10 of FC in Figure 1C were validated by real-time quantitative RT-PCR (Table S1, Figure S1). Of these, *lnc-SLC15A1-1* expression was significantly (*p* < 0.01) increased in HUVECs in HG + IS treatment by real-time quantitative RT-PCR (Figure 1E). Therefore, we focused on *lnc-SLC15A1-1* in the following experiments.

### 2.2. General Characterization of lnc-SLC15A1-1

**Figure 2:**
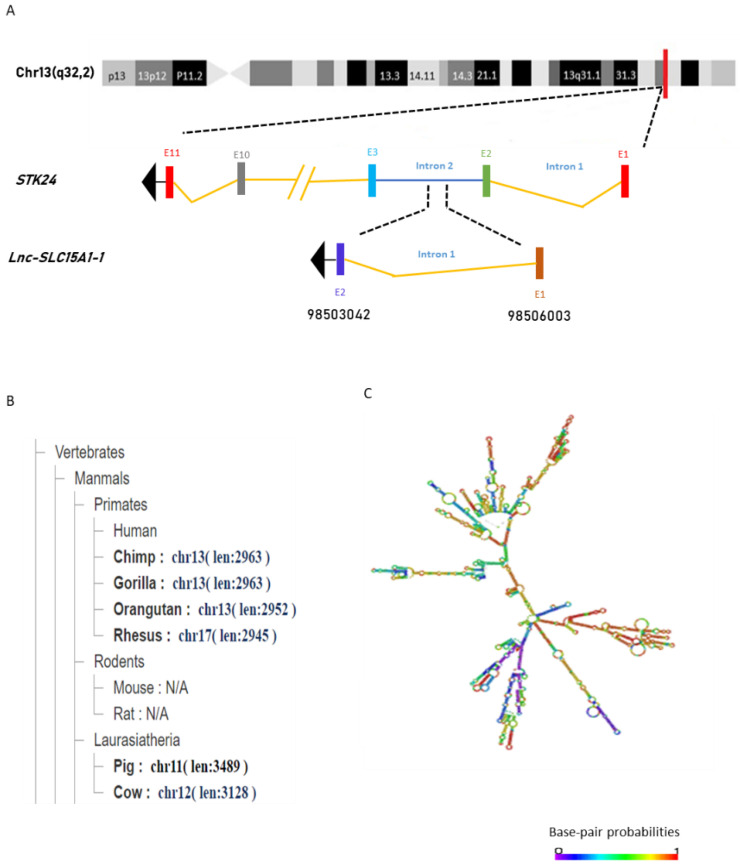
General characterization of *lnc-SLC15A1-1*. (**A**) Schematic representation of *lnc-SLC15A1-1*. (**B**) Phylogenetic tree of *lnc-SLC15A1-1* across species. (**C**) The predicted secondary structure of *lnc-SLC15A1-1*. The minimum free energy structure was colored by base-pairing probabilities using the RNAfold web server (http://rna.tbi.univie.ac.at/cgi-bin/RNAWebSuite/RNAfold.cgi, accessed on 4 May 2020).

From the information in the NONCODE database (http://www.noncode.org/, accessed on 25 December 2019), we found that *lnc-SLC15A1-1* is located on chromosome 13 (q32.2) and transcribed from part of *STK24*’s intron 2 in the anti-sense strand. It consists of 1795 nucleotides, with two exons (Figure 2A). Phylogenetic analysis showed that *lnc-SLC15A1-1* is well conserved across species, including primates and laurasiatheria, but is located in different chromosomes. *Lnc-SLC15A1-1* in chimpanzees, gorillas, and orangutans is located on chromosome 13, like humans, but is on chromosome 17 in Rhesus monkeys, chromosome 11 in pigs, and chromosome 12 in cows (Figure 2B). However, the function of *lnc-SLC15A1-1* has not been mentioned in the literature. Figure 2C shows the predicted minimum free energy secondary structure of *lnc-SLC15A1-1*, which was provided by RNAfold network (http://rna.tbi.univie.ac.at//cgi-bin/RNAWebSuite/RNAfold.cgi, accessed on 4 May 2020). This secondary structure is composed of highly probable base pairs and annotated in different colors representing the probability of base pairing.

### 2.3. Identification of Genes Regulated by Lnc-SLC15A1-1 in HUVECs by Microarray Analysis

**Figure 3:**
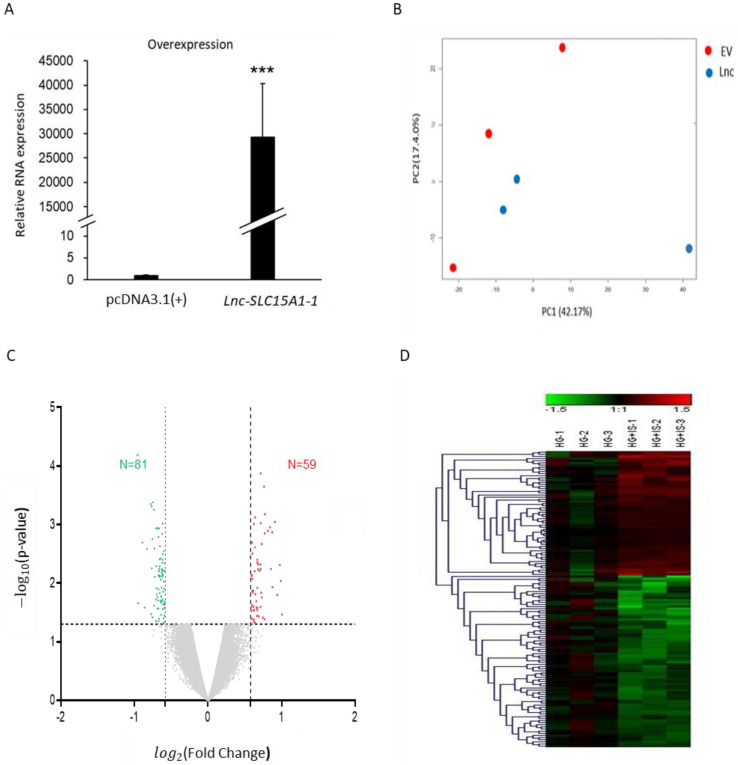
Identification of genes regulated by *lnc-SLC15A1-1* in HUVECs by microarray analysis. (**A**) Relative expression levels of *lnc-SLC15A1-1* by quantitative RT-PCR. HUVECs were transfected with 1.5 μg of *lnc-SLC15A1-1* expression plasmids (pcDNA3.1(+)-*lnc-SLC15A1-1*). The *lnc-SLC15A1-1* RNA expression levels were normalized to *18S* rRNA (*** *p* < 0.001). (**B**) Principal component analysis. HUVECs were transfected with empty vector (EV; red spots) or *lnc-SLC15A1-1* expression plasmids (Lnc; blue spots). The expression levels were analyzed by Affymetrix Clariom™ S Assay (*n* = 3). The expression levels of total mRNAs were used for plotting PCA. (**C**) Volcano plot of differentially expressed genes (DEGs) in HUVECs over-expressing *lnc-SLC15A1-1*. DEGs with fold change ≥1.5 or ≤−1.5 and *p*-value < 0.05 are labeled as red or green spots, respectively. (**D**) Heatmap with hierarchical clustering. Red color indicates upregulated genes; green color indicates downregulated genes.

To explore the function of *lnc-SLC15A1-1*, HUVECs were transfected with pcDNA3.1(+)-*lnc-SLC15A1-1*. The relative expression levels of *lnc-SLC15A1-1* in HUVECs significantly (*p* < 0.001) increased, as determined by quantitative RT-PCR normalized to *18S* rRNA (Figure 3A). Microarrays were used to identify the downstream genes regulated by *lnc-SLC15A1-1* (GSE185363). PCA showed that the distribution of HUVECs overexpressing *lnc-SLC15A1-1* was different than that of those expressing the EV (empty vector) control, indicating the different mRNA expression patterns between groups (Figure 3B). By the criteria of FC ≥1.5 or ≤−1.5 and *p*-value <0.05, there were 150 differentially expressed genes (DEGs) as a result of increased *lnc-SLC15A1-1* expression, 59 of which showed significant upregulation and 81 of which showed downregulation (Figure 3C,D).

### 2.4. Ingenuity Pathway Analysis of Differentially Expressed Genes Regulated by lnc-SLC15A1-1

**Figure 4:**
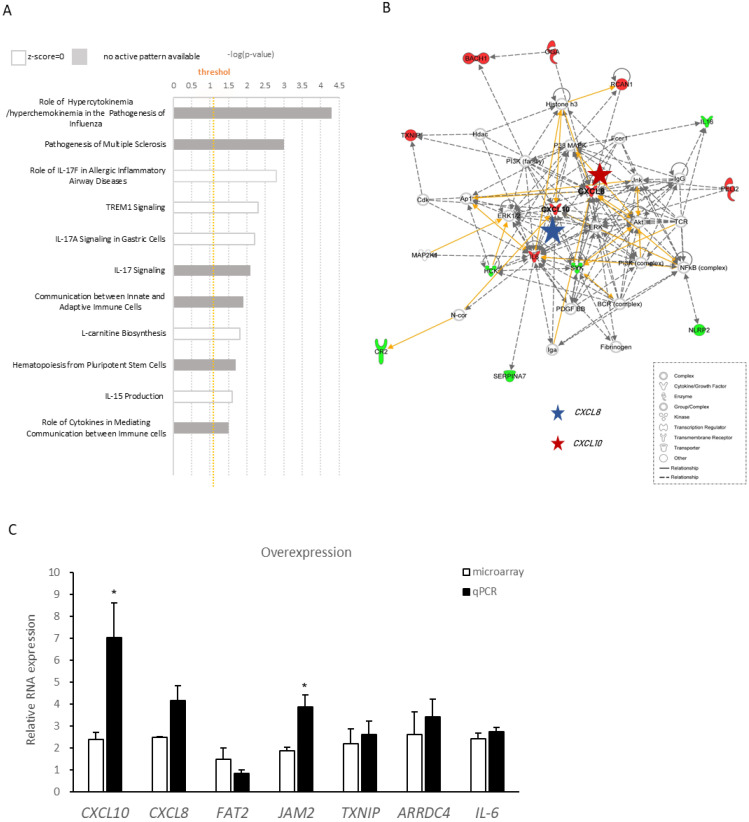
Ingenuity Pathway Analysis of differentially expressed genes regulated by *lnc-SLC15A1-1*. (**A**) Canonical pathways of differentially expressed genes regulated by *lnc-SLC15A1-1*. Vertical orange line indicates the default threshold. The gray bars indicate no activity pattern. (**B**) Representative network of downstream genes regulated by *lnc-SLC15A1-1*. Red and blue stars indicate *CXCL10* and *CXCL8*, respectively. Green color indicates downregulation, and red color indicate upregulation. Solid lines represent direct interaction (yellow), and dashed lines represent indirect interaction. Various shapes representing the functional class of the gene product. (**C**) Quantitative RT-PCR analyses of top 10 differentially expressed genes identified by microarray. Loading control: *GAPDH*. Experiments were repeated in triplicate, and the results are the mean ± SDs. (* *p* < 0.05).

To further evaluate the function *lnc-SLC15A1-1*, IPA was performed to identify the signaling pathways in which DEGs were involved. As a result, the most significant canonical pathway was “role of hypercytokinemia/hyperchemokinemia in the pathogenesis of influenza” (Figure 4A), suggesting that *lnc-SLC15A1-1* may be involved in the hyperactivation of cytokines and chemokines. By further analyzing the molecular networks of downstream genes regulated by *lnc-SLC15A1-1*, we were interested in two hub genes encoding chemokines, *CXCL8* (blue star) and *CXCL10* (red star), because they were consistent with the canonical pathways of chemokine and cytokine hyperactivation (Figure 4B). Seven of the top 10 DEGs identified by microarray were chosen for RT-PCR validation (Table S2). As shown in Figure 4C, the results were compatible with the trends of gene expression by microarray. The two molecules with highest fold changes were *CXCL10* and *CXCL8* (Figure 4C).

### 2.5. Upregulation and Secretion of CXC10 and CXCL8 in HUVECs by lnc-SLC15A1-1

**Figure 5:**
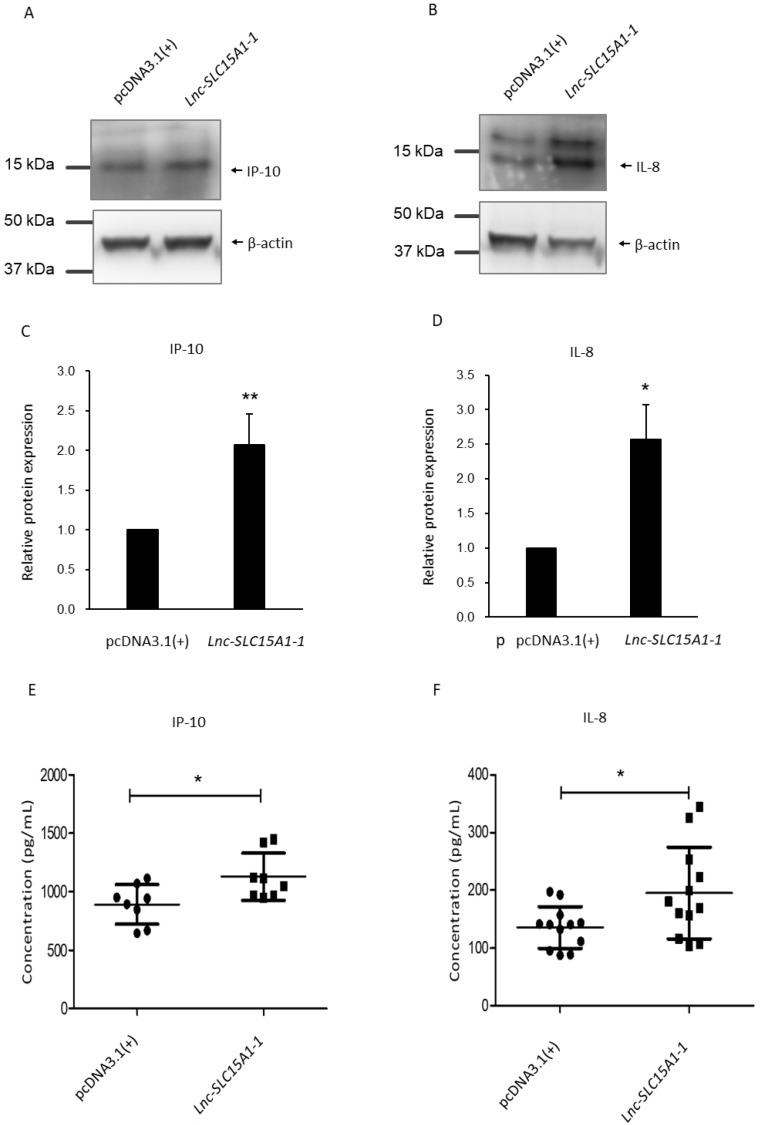
Upregulation of IP-10 and IL-8 in HUVECs overexpressing *lnc-SLC15A-1*. (**A**,**B**) Total lysates from HUVECs transfected with 1.5 μg of *lnc-SLC15A1-1* expression plasmid or empty vector (pcDNA3.1(+)) were analyzed by Western blotting. Loading control: β-actin. (**C**,**D**) Quantitative results of Western blotting. Analysis and quantification of Western blotting results of IP-10 and IL-8. Data are the means ± SDs (*n* = 3; * *p* < 0.05, ** *p* < 0.01). (**E**,**F**) Enzyme-linked immunosorbent assay (ELISA) of IP-10 and IL-8 in conditioned medium. Culture medium of HUVECs with/without overexpression of *lnc-SLC15A1-1* was collected, followed by ELISA application. All data shown are the means ± SDs (* *p* < 0.05).

Since *CXCL10* and *CXCL8* were upregulated in HUVECS overexpressing *lnc-SLC15A1-1*, their protein levels were examined by western blotting. *CXCL10* encodes IP-10 [33], and *CXCL8* encodes IL-8 [34]. As shown in Figure 5A–D and Figure S2, IP-10 and IL-8 were significantly (*p* < 0.05) increased in HUVECs overexpressing *lnc-SLC15A1-1*. Since chemokines can be secreted to the extracellular space to attract immune cells [35], IP-10 (Figure 5E) and IL-8 (Figure 5F) were also detected in the conditioned cell culture medium by an ELISA kit. Indeed, the protein amounts of IP-10 and IL-8 significantly (*p* < 0.05) increased in the medium of cells expressing *lnc-SLC15A1-1*, indicating that *lnc-SLC15A1-1* promotes the secretion of these proteins.

### 2.6. Lnc-SLC15A-1 Distribution in HUVECs

**Figure 6:**
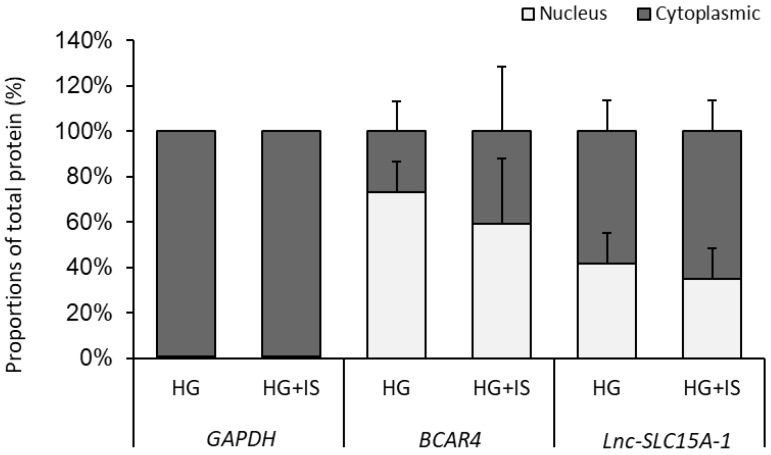
*Lnc-SLC15A-1* distribution in HUVECs. Cytoplasmic and nuclear RNA were fractionated in HUVECs overexpressing *lnc-SLC15A1-1* under HG and HG + IS treatment. Relative amounts of RNA were normalized to the total amount of RNA and detected by quantitative RT-PCR. Nuclear markers: *BCAR4* snRNA. Cytoplasmic marker: *GAPDH*. Data shown are the means ± SDs (*n* = 8).

To investigate the mechanism by which *lnc-SLC15A1-1* upregulates *CXCL10* and *CXCL8*, we examined the distribution of *lnc-SLC15A1-1* in HUVECs overexpressing *lnc-SLC15A1-1* and treated with HG or HG + IS by cytoplasmic and nuclear fractionation. Around 60–70% of *lnc-SLC15A1-1* was located in the cytoplasm (Figure 6), indicating that *lnc-SLC15A1-1*’s upregulation of *CXCL10* and *CXCL8* may be via miRNA sponging, i.e., binding and sequestration of specific miRNA transcripts.

### 2.7. MiRNA Sponge Function of lnc-SLC15A1-1

**Figure 7:**
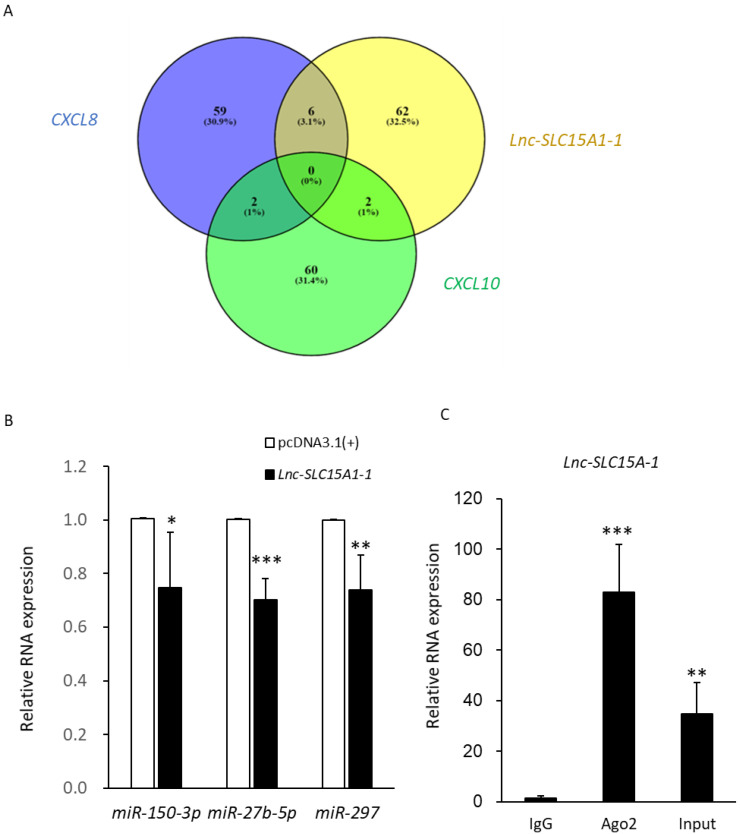
*Lnc-SLC15A1-1* functions as a miRNA sponge. (**A**) Venn diagram of miRNAs predicted to interact with *lnc-SLC15A1-1*, *CXCL10*, and *CXCL8*. MiRNAs were predicted by miRDB (http://mirdb.org/index.html, accessed on 17 December 2020). (**B**) Relative expression levels of three predicted miRNAs. The expression levels of miRNAs in HUVECs overexpressing *lnc-SLC15A1-1* or empty vector (pcDNA3.1(+)) were detected by quantitative RT-PCR and normalized to *U6*. Data are the means ± SDs (* *p* < 0.05, ** *p* < 0.01, and *** *p* < 0.001). (**C**) RNA immunoprecipitation (RIP) analysis of *lnc-SLC15A1-1* using antibody against Argonaute-2 (AGO2) in HUVECs. The RIP enrichment of the AGO2-associated lncRNA was measured by quantitative RT-PCR and normalized to *U6*. The relative fold enrichment was calculated as compared to the IgG group. Data are the means ± SDs (*n* = 5 and ** *p* < 0.01, *** *p* < 0.001).

In order to identify the miRNAs interacting with *lnc-SLC15A1-1* and its target genes *CXCL10* or *CXCL8*, the miRNA candidates were predicted by miRDB (MicroRNA Target Prediction Database). As shown in Table 1, there were two miRNAs targeting *CXCL10* and six miRNAs targeting *CXCL8* that were also predicted to bind to *lnc-SLC15A1-1*. None of the miRNAs targeted all three molecules (*CXCL10*, *CXCL8*, and *lnc-SLC15A1-1)* (Figure 7A). Three miRNAs, *miR-27b-5p*, *miR-297*, and *miR-150-3p*, were validated by RT-PCR (Table 1, Tables S3 and S4), and their expression levels were significantly (*p* < 0.05) decreased in HUVECs overexpressing *lnc-SLC15A1-1* (Figure 7B).

To confirm the function of *lnc-SLC15A1-1* as a miRNA sponge, we performed RIP (RNA immunoprecipitation) using an antibody against AGO2 protein, a hub of RNA-induced silencing function, in order to evaluate the interaction with *lnc-SLC15A1-1*. The amount of *lnc-SLC15A1-1* IgG-linked beads as control was significantly increased in whole cell RNAs (*p* < 0.01). It was found that *lnc-SLC15A1-1* was present at higher levels in AGO2-associated beads than in lgG-linked beads (*p* < 0.001), as measured by RT-PCR (Figure 7C). This shows that *lnc-SLC15A1-1*, by acting as a miRNA sponge, could upregulate *CXCL10* and *CXCL8* gene and protein expression.

## 3. Discussion

In this study, we discovered a novel pathway by which *lnc-SLC15A1-1* upregulates the chemokines *CXCL10* and *CXCL8* via its function as a miRNA sponge in HUVECs subjected to endothelial injury. This novel pathway may be a contributing factor responsible for inflammation in the endothelium, which leads to the high incidence of cardiovascular events in DKD compared with DM.

CVD is the leading cause of death in DM and CKD, and both are risk factors for CVD. Because of the link between kidney function and cardiovascular health, there is no doubt that DKD would increase CVD events more than DM. Although a large number of pathophysiological studies have elucidated the link between DM or CKD and CVD, there has been little research about the mechanism of worsening of the cardiovascular system in DKD. Studies have indicated that uremic toxins activate harmful signal pathways, leading to overexpression of proinflammatory [36] and prothrombotic proteins [37], which cause atherosclerosis. Therefore, endothelial function and the pathways modulated by uremic toxins may be potential targets for novel therapies. To further elucidate these pathways, we conducted this study using HUVECs subjected to HG and HG + IS to mimic DM and DKD conditions in human endothelium cells. From the literature, the IS serum level in uremic patients is around 0.07–0.2 mM [38,39]. We used a concentration of 0.1 mM IS in this study to represent damage to the vascular system by CKD.

Endothelial dysfunction is an early marker of CVD frequently observed both in CKD and DM patients [40]. A study, comparing the inflammation status in DM with/without nephropathy, demonstrated that plasma levels of proinflammatory monocytes as well as circulatory inflammatory mediators, such as PAI-1, syndecan-1, VEGF, IL-1β, IL-1Ra, and CCL4, increased in nephropathy subjects [41]. Moreover, peripheral blood mononuclear cells from DKD patients co-cultured with primary endothelial cells increased soluble ICAM and PAI-1 expression [41]. These results suggested the importance of inflammation and endothelial dysfunction in DKD.

Recent evidence suggested that the accumulation of certain uremic toxins, such as IS, was responsible for the dysfunction of immune cells in CKD [42]. For example, IS levels in CKD patients were associated with monocyte activation, intensified inflammatory process, augmented oxidative stress, and disturbed hemostatic system, which contributed to the development of atherosclerosis [43]. IS could accelerate proinflammatory macrophage activation and further induce vascular inflammation in CKD [44].

Numerous studies have also reported that IS had detrimental effects on endothelial cells and was described as an endotheliotoxin to induce multiple processes such as pro-inflammatory, pro-oxidative, AhR activation and endothelial dysfunction [45]. Although plasma levels of IS were found to be associated with various cardiovascular diseases in DKD, the causal relationship is still unclear, and data regarding endothelial dysfunction in DM/CKD patients are rare.

Given that endothelial cells incubated with uremic serum significantly increased the levels of inflammatory biomarkers of IL-6, IL-8, MCP-1, and PAI-1 [46], we aimed to evaluate the impact of IS on the high glucose endothelial cells to clarify the injury mechanism of DM/CKD patients’ endothelium. Our results showed that inflammation was the important mechanism of endothelium dysfunction when concomitantly exposed to IS and high glucose. IP-10 and IL-8 seemed to be the inflammatory molecules to activate the process. These results were consistent with past literature, showing IP-10 expression increased in patients with advance CKD [47] and that IS induced oxidative stress by upregulation of IL-8 in the endothelial cells to recruit neutrophils and initiate inflammation [48,49]. However, more experiments in vivo are still warranted.

Due to the development of deep sequencing technologies, a vast number of non-protein-coding RNAs, which were previously regarded as junk DNA, were found to possess important biological functions that regulate cellular homeostasis. Many studies have found that lncRNAs such as *MALAT1*, *ANRIL/CDKN2BAS*, *HI-LNC25*, and *KCNQ1OT1* are closely associated with type 2 diabetes susceptibility genes, which might affect their expression [25,26,27]. Moreover, *MANTIS* was shown to relieve uremic toxin p-cresyl sulfate-induced HUVEC injury [23].

Recent findings have also indicated that the lncRNA–miRNA–mRNA axis is a novel regulatory mechanism modulating DM. It was reported that the lncRNA *NEAT1*/*miR-34c*/*NLRP3* axis regulates pyroptosis activation and that the lncRNA *MEG3*/*miR-181a*/Egr-1/TLR4 signaling pathway promotes fibrosis and the inflammatory response, both of which mediate the progression of diabetic nephropathy [50,51]. Moreover, the lncRNA *AK139328*/*miR-204-3p*/Atg7, Atg5, and LC3-II/LC3-I axis influences autophagy upon myocardial ischaemia/reperfusion injury in diabetic mice [52]. In this study, we discovered the overexpression of *lnc-SLC15A1-1* activation in HUVECs treated with HG + IS and the subsequent upregulation of *CXCL10* and *CXCL8* genes and proteins, which might explain the connection between endothelial injury and atherosclerosis.

Our results highlighted that the chemokines IP-10 and IL-8 were upregulated in HUVECs cultured in HG + IS, which simulated the injury of vascular endothelial cells in DKD patients. Numerous studies have corroborated that a systemic inflammatory reaction and oxidative stress are two major mechanisms of vascular injury in patients with CKD or DM [53,54]. Chemokines are chemotactic cytokines with low molecular weight that help attract immune cells to the site of inflammation, which is one of the earliest events in atherosclerosis [55,56]. IP-10 and IL-8 could be secreted from various immune cells and endothelial cells into the extracellular space, inducing the migration of cells to the site of inflammation, where they are proven participants in atherosclerotic lesion formation [8,57]. It has been reported that the serum levels of IP-10 and IL-8 in diabetic patients are higher than in healthy subjects [58,59]. In addition, research revealed that *CXCL10* and *CXCL8* are generated by stimulation with the uremic toxin IS [6,60]. In our investigation, mRNA and protein levels of *CXCL10* and *CXCL8* in HUVECs overexpressing lnc-SLC15A1-1 were escalated and actively secreted to the extracellular space as proteins significantly increased in conditioned medium (Figure 5E,F).

Accumulated data have shown that numerous inflammatory molecules are engaged in all phases of CVD. It is well known that multiple adhesion molecules and selectins are upregulated to facilitate the binding, rolling, and transmigration of inflammatory cells in the injured area [61]. The molecules of the Nod-like receptor protein 3 (NLRP3) inflammasome in macrophages propagate the inflammatory process. Moreover, a variety of cytokines, such as IL-1β, IL-6, IL-18, nuclear factor kappa B (NF-kB), and TNF-α, are involved in the activation of the inflammatory cascade within the vessel wall [62,63,64,65]. However, the key elements in DKD-induced CVD among all these inflammatory factors are still unclear. Our results suggest that DKD patients’ higher rate of cardiovascular events may result, in part, from upregulation of *CXCL10* and *CXCL8* by *lnc-SLC15A1-1*, but further research for validating the in vitro data in animals and clinical patients is still needed.

Moreover, we identified three miRNAs, *miR-150-3p*, *miR-297*, and *miR-27b*, targeting *CXCL10* and *CXCL8*, for which there was no previous information regarding an association with DM or CKD-related diseases or a relationship with *lnc-SLC15A1-1*. In literature reviews, the functions of *miR-150-3p* and *miR-297* were related to cancer [66,67]. Moreover, *miR-27b-5p* exerted biological effects on HUVECs, fibroblasts, and breast cancer cells in previous reports [68,69,70]. In order for the functions of these three miRNAs to be clarified, advanced studies to evaluate their effect on DM and its complications are necessary.

The new axis discovered in this study might play a role in the heightened rate of CVD in DKD and could be a therapeutic target in future clinical applications. Nevertheless, there were some limitations of this study. First, when renal function deteriorates, a variety of uremic toxins accumulate in the body. In our study, we only tested one kind of uremic toxin—IS, which was proven to exert toxicity on endothelial cell. Thus, our findings might explain only one aspect of the induction of CVD by DKD. In order for the causal relationship of IS and endothelial injury among various uremic toxins in DKD to be elucidated, modulation of the amount of IS in an animal model of DM with renal failure will further confirm the conclusions of this study, such as decreasing IS synthesis via handling gut microbiota [71,72], reducing indole absorption by spherical carbon adsorbent AST-120 [73], increasing IS serum level by oral or intravenous extra IS supplement, or inhibiting the function of organic anion transporters 1 and 3 (OAT1/3) [74]. Second, our data regarding the important role of *lnc-SLC15A1-1*; chemokines *CXCL10* and *CXCL8*; and miRNAs *miR-150-3p*, *miR-27b-5p*, and *miR297* are derived from in vitro studies. It must be noted that *lnc-SLC15A1-1* and *miR-150-3p*, *miR-27b-5p*, and *miR-297* have rarely been noted in past research, and thus the function of these molecules should be further evaluated. Third, miRNAs targeting *CXCL10* and *CXCL8* were predicted by the miRDB network, and their regulatory relationship also needs to be further verified.

## 4. Conclusions

In this study, we assessed aspects of *lncRNA* regulation and function in DKD using in vitro cell models. The results suggested that upregulated lnc-SLC15A1-1 had a devastating impact on HUVEC proliferation via induction of CXCL10 and CXCL8 expression. Our results suggest that *lnc-SLC15A1-1* increases chemokine expression via sponging the miRNAs for these transcripts, and this pathway might be a novel therapeutic target to treat CVD in DKD. Current strategies for the treatment of DKD and its complications can only delay its onset and slow progression, and a significant proportion of patients still suffer from atherosclerosis. Efficacious and specific therapies for DKD have yet to be discovered, and our findings might supply another treatment target in the future. However, it is still necessary to clarify the characteristic features of the *lnc-SLA15A1-1/miR-150-3p*, *miR-27b-5p/CXCL10*, and *lnc-SLA15A1-1/miR-297/CXCL8* pathways before using them as therapeutic targets.

## 5. Materials and Methods

### 5.1. Cell Culture and Treatments

HUVECs were purchased from Lonza Walkersville, Inc. (Clonetics™ Endothelial Cell System; cat. no. C2519A). Cells were cultured in EGMTM-2 Endothelial Cell Growth Medium-2 BulletKitTM (cat. no. CC-3162, Lonza, Houston, TX, USA), which contained EBMTM-2 Basal Medium (cat. no. CC-3156, Lonza, Houston, TX, USA) and EGMTM-2 SingleQuots^TM^ Supplements (cat. no. CC-4176, Lonza, Houston, TX, USA), in addition to 1% penicillin–streptomycin–amphotericin solution (Biological Industries, Beit-Haemek, Israel), and incubated at 37 °C in a humidified atmosphere with 5% CO_2_. HUVECs at passages 5–8 were used for the in vitro studies.

Cells from the fifth passage were incubated in 30 mM D-glucose (high glucose, HG) or 30 mM D-glucose treated with 0.1 mM IS (HG + IS) (cat. no. I3875, Sigma-Aldrich, Germany) for 96 h. In some experiments, HUVECs were transfected with 1.5 μg of a *lnc-SLC15A1-1* expression plasmid (pcDNA3.1(+)-lnc-SLC15A1-1) or empty vector (EV) as a control by employing the TransIT-X2 delivery system (cat. no. MIR6000, Mirus Bio, Madison, WI, USA). The transfection efficiency of HUVECs was assayed by quantifying *lnc-SLC15A1-1* transcripts by RT-PCR. Each experiment had HUVECs from the same batch and the same passage and was repeated 3–4 times.

### 5.2. Cell Proliferation Assay

HUVECs were first cultured in a 6 cm dish. Then, the fifth passage cells were re-seeded in a 96-well microplate at a density of 2500 cells per well and incubated in HG or HG + IS for 4 days with medium changed every day. Cell proliferation was measured by 3-(4,5-dimethylthiazol-2-yl)-2,5-diphenyl tetrazolium bromide (MTT) (EMD Biosciences, San Diego, CA, USA) assays on days 0, 1, 2, 3, and 4 (*n* = 3). Each well received a 100 μL mixture consisting of 10 μL of MTT solution with 90 μL of RPMI medium and was then incubated for 2 h. The absorbance was measured at 570 nm. The cell growth ratio was expressed as relative absorbance compared with day 0.

### 5.3. RNA Extraction, Reverse Transcription, and Quantitative RT-PCR

Total RNA was extracted from cultured cells using NucleoZOL reagent (cat. no. 740404.200, Machery-Nagel, Germany) according to the manufacturer’s protocol. One microgram of total RNA was reverse-transcribed by a High-Capacity cDNA Reverse Transcription Kit (Applied Biosystems, Carlsbad, CA, USA). Transcript cDNA of 2.5% used as template was added into each well of 96 plates mixed with primers shown in Table S1 and FastStart Universal SYBR Green Master (Roche, Switzerland) for performing real-time PCR. Finally, the reaction was executed with an ABI Step One plus system (Applied Biosystems, Carlsbad, CA, USA). Each reaction was duplicated, and data were normalized to *GAPDH*. Quantitative RT-PCR was executed by a Step One Plus Real-Time PCR System (Thermo Fisher Scientific, Waltham, MA, USA). The mRNA expression levels were normalized to *GAPDH* transcripts and relative mRNA levels were measured using the 2^−ΔΔCT^ method.

### 5.4. RNA Sequencing Library Preparation

RNA concentration and quality were determined using a Qubit Fluorometer (Thermo Fisher Scientific, Waltham, MA, USA) and an Agilent 2100 Bioanalyzer (Agilent TechnologiesInc., Wood Dale, IL, USA), which calculates an RNA integrity number (RIN). Total RNA with A_260_/A_280_ = 1.8–2.0 and RIN >8.0 was selected. Intact RNA samples were normalized to 100 ng/μL with DEPC-treated H_2_O. A total amount of 1 µg RNA was purified. The ribosomal RNAs (rRNA) were eradicated from total RNA using a Ribo-zero rRNA removal kit (cat. no. 20278677, Illumina Inc., San Diego, CA, USA). After purification, the RNAs were fragmented into small pieces by using divalent cations with elevated temperature. The cleaved RNA fragments were copied into first strand cDNA using reverse transcriptase and random primers, followed by second strand cDNA synthesis using DNA Polymerase I and RNase H. cDNA fragments went through an end repair process with addition of a single “A” base and ligation of the adapters. The products were then purified and enriched with PCR to create the final cDNA library. Paired-end reads from each cDNA library were obtained using a NextSeq500 system (Illumina Inc., San Diego, CA, USA).

### 5.5. Screening and Cluster Analysis of Differentially Expressed lncRNAs

Differences in the expression of the lncRNAs were calculated on the basis of the trimmed mean of M-values method [75]. LncRNAs with similar expression patterns were analyzed using the hierarchical complete linkage clustering method in R software (R package, version 3.4.3; www.r-project.org/, accessed on 17 October 2019). Significant differences in lncRNA expression levels were determined on the basis of a *p*-value cut-off of 0.05 and a minimum fold change (FC) of 1.5 using DEseq software package version 1.20.0 [76]. The differentially expressed lncRNAs were replaced by log_2_ values (data values), and the Euclidean distance was calculated. The results were further analyzed using R packages; specifically, the “heatmap 2” function of the “gplots” package, which was used to draw heat maps of the differentially expressed lncRNAs.

### 5.6. Plasmid DNA Construction and Transfection

To overexpress *lnc-SLC15A1-1* in HUVECs, we constructed plasmids by the BioMed Resource Core of the 1st Core Facility Lab, National Taiwan University College of Medicine (NTU-CM) (Taipei, Taiwan). The expression plasmid (pcDNA3.1(+)-*lnc-SLC15A1-1*) was constructed by inserting the full length *lnc-SLC15A1-1* sequence (1795 bp) into the pcDNA3.1(+) expression vector (Addgene, Watertown, MA, USA). HUVECs were transfected with the expression vectors by TransIT-X2 transfection reagent (cat. no. MIR6000, Mirus Bio, Madison, WI, USA).

### 5.7. Isolation and Amplification of Total RNA for Gene Expression Profiling by Microarrays

Total RNAs extracted from the HUVECs infected with lentivirus expressing *lnc-SLC151A-1* or EV were isolated by using NucleoZOL reagent (Machery-Nagel, Germany) and treated with DNase I following the manufacturer’s instructions. RNA was amplified (in vitro transcription), and biotin-labeled single-stranded cDNA was generated according to the manufacturer’s instructions. Single-stranded cDNA was generated from the amplified cRNA with the GeneChip™ WT PLUS Reagent Kit and then fragmented and labeled with the GeneChip™ WT Terminal Labeling Kit. Samples were hybridized with Clariom™ S Assay human arrays and were scanned using the GeneChip™ Scanner 3000 7G at the National Health Research Institutes (NHRI) Microarray Core Facility.

Affymetrix GeneChip Command Console Software v4.0 (AGCC 4.0, Affymetrix^®^) and Transcriptome Analysis Console (TAC) Software v4.0.1 were used with default analysis settings, and expression values were processed with the signal space transformation and robust multichip average methods, which included log_2_ transformation and quantile normalization.

Genes were considered differentially expressed if the adjusted *p*-value was less than 0.05 and the mean level of expression was greater than 1.5-fold. The genes that were identified from the microarray analyses were verified by quantitative RT-PCR assays with the primer from Table S2.

### 5.8. Analysis of Functional Enrichment and the lncRNA-Gene Network

The genes with altered expression profiles were imported into the Ingenuity Pathway Analysis Tool (IPA Tool; Ingenuity^®^ Systems, Redwood City, CA, USA; http://www.ingenuity.com, accessed on 25 July 2020). Differentially expressed genes were mapped to genetic networks in the Ingenuity database in order to identify the biological networks and functional pathways involved. Genes with *p*-values less than 0.05 and expression greater than 1.5-fold were uploaded.

In the graphical network, molecules were represented as nodes and the biological relationship between two nodes showed as an edge (line). Hypergeometric tests with the Benjamini and Hochberg false discovery rates were performed using the default parameters in order to adjust the Q-value.

### 5.9. Western Blotting

Due to the low molecular weight of chemokines IP-10 and IL-8, the protein lysates extracted from the HUVECs overexpressing *lnc-SLC15A1-1* were separated by a Tris-Tricine SDS-PAGE gel electrophoresis system (cat. no. ab119197, Abcam, UK). For the Western immunoblotting assay, the target protein was transferred to a 0.22 μm polyvinylidene difluoride membrane (Bio-Rad Laboratories, Hercules, CA, USA) following the manufacturer’s instructions. The membranes were blocked in Lightning Blocking Buffer (Arrowtec, Taiwan) for 5 min and then hybridized to primary antibodies against IP-10 (cat. no. AHP782, Bio-Rad Laboratories, Hercules, CA, USA) or IL-8 (cat. no. ab52612, Abcam, UK). After immunoblotting, the membranes were washed in 0.05% Tris-buffered saline (Omics Bio, Taiwan) with Tween 20 and reacted with horseradish peroxidase-conjugated goat anti-mouse IgG (cat. no. C04001, Croyez Bioscience, Taiwan). The protein bands were visualized using an enhanced chemiluminescence system (cat. no. GTX14698, GeneTex, Irvine, CA, USA). β-actin was used as the loading control (cat. no. A5441, Sigma-Aldrich, Germany).

### 5.10. ELISA

IP-10 and IL-8 in the supernatant of cultured HUVECs transfected with pcDNA3.1(+)-*lnc-SLC15A1-1* or EV were measured with an ELISA IP-10 kit (cat. no. 439904, BioLegend, San Diego, CA, USA) or an ELISA IL-8 kit (cat. no. 431504, BioLegend, San Diego, CA, USA) according to the manufacturer’s instructions.

### 5.11. Nuclear Cytoplasmic RNA Fractionation

HUVECs (6.5 × 10^5^ cells per dish) were plated in a 6 cm dish. Cells were cultured under HG and HG + IS conditions after seeding overnight. RNA was isolated from HUVECs growing in a monolayer using a Cytoplasmic and Nuclear RNA Purification Kit (cat. no. 21000, Norgen Biotek Corp., Thorold, ON, Canada) according to the manufacturer’s instructions. Briefly, HUVECs were lysed and then centrifuged. The cytoplasmic RNA was contained in the supernatant, while the nuclear RNA was in the pellet. RNA samples were separated in spin-columns. After washing and centrifugation steps, purified cytoplasmic and nuclear RNA was eluted from the columns. The procedure was performed in duplicate.

### 5.12. MicroRNA Extraction, Reverse Transcription, and Quantitative RT-PCR

Total RNA was extracted from HUVECs with/without *lnc-SLC15A1-1* overexpression by NucleoZOL reagent (cat. no. REF 740404.200, Machery-Nagel, Germany). RNA concentration was measured by an ND-1000 NanoDrop spectrophotometer (Thermo Fisher Scientific, Waltham, MA, USA). For the Universal Probe Library probe assay, miRNA (1 μg) was reverse-transcribed into cDNA with 25 nM stem loop RT primer by SuperScript™ II reverse transcriptase (Invitrogen, Carlsbad, CA, USA) for targeted miRNA detection. The RT stem loop primer cDNA sequences described in Table S3 were used as the template to design the RT-PCR primers. Quantitative RT-PCR was performed with forward primers outlined in Table S4 and a universal primer (reverse: 5′-GTGCAGGGTCCGAGGT-3′) mixed well with SYBR Green (cat. no. FPT-BB05, Biotools, Taiwan). Finally, the reactions were executed on an ABI Step One plus system (Applied Biosystems, Carlsbad, CA, USA). The miRNA expression levels were normalized to *U6* RNA using the 2^−ΔΔCT^ method.

### 5.13. RNA Immunoprecipitation (RIP)

RIP was performed using the Magna RIP^TM^ RNA-Binding Protein Immunoprecipitation Kit (cat. no. 17-700, Merck Millipore, Burlington, MA, USA) according to the manufacturer’s instructions.

HUVECs (4 × 10^7^ cells) were seeded in 15 cm plates. After seeding overnight, cells total RNA was extracted by RIP lysis buffer (cat. no. 17-700, Merck Millipore, Burlington, MA, USA) with protease and RNase inhibitor cocktail. The RNA lysis sample and Argonaute-2 protein (AGO2) bound beads were mixed in immunoprecipitation buffer overnight. The immunoprecipitation buffer was composed of RIP wash buffer, 0.5 M ethylenediaminetetraacetic acid, and RNase inhibitor. Then, the supernatant was removed and mixed with proteinase K and 10% SDS buffer at 55 °C for 30 min. The supernatant was taken out and purified. The amount of captured RNA was measured by quantitative RT-PCR.

## Figures and Tables

**Table 1:** MicroRNAs predicted to interact with *lnc-SLC15A1-1* and target *CXCL10* and *CXCL8*.

	*CXCL10/lnc-SLC15A1-1*	PCR Validation	*CXCL8*/*lnc-SLC15A1-1*	PCR Validation
MicroRNA	*miR-27b-5p* *miR-297*	○ ○	*miR-150-3p* *miR-513a-3p* *miR-3606-3p* *miR-513c-3p* *miR-10523-5p* *miR-567*	○ X X X

○: miRNAs could be validated; X: miRNAs could not be validated.

## Data Availability

RNA-seq and microarray raw data are uploaded to GEO dataset with accession numbers of GSE185598 and GSE185363, respectively.

## Supplementary Materials

The following are available online at https://www.mdpi.com/article/10.3390/toxins13120873/s1, Figure S1: Primer tests of selected lncRNAs and the results of RT-PCR. Figure S2: Original Western Blot gel of IP-10 and IL-8. Table S1: Primers of lncRNAs. Table S2: Primers of microarray confirmed mRNAs. Table S3: RT stem loop primer sequences. Table S4: Primers of miRNAs.
