# Supplementary material for: Indoxyl Sulfate Elevated Lnc-SLC15A1-1 Upregulating CXCL10/CXCL8 Expression in High-Glucose Endothelial Cells by Sponging MicroRNAs
Source: Toxins (Basel). 2021 Dec 7;13(12):873. doi: 10.3390/toxins13120873 (PMC8709190; doi:10.3390/toxins13120873)
Supplement: Supplementary file 1 [file toxins-13-00873-s001.zip › toxins-1443772-supplementary.pdf]

Supplement

Indoxyl Sulfate Elevated *Lnc-SLC15A1-1* Up-regulating CXCL10/CXCL8 Expression in High Glucose Endothelial Cells by Sponging MicroRNAs

Table S1. Primers of lncRNAs.

Transcript ID	Primer sequence 1	Primer sequence 2	Primer sequence 3
NONHSAT 238235.1	F- CCTCTTGCCCTGTTGAGAGC<br>R- TCCTTTCGGGGTTCAGCAAA	F- GTCCCGCTCCACTTCATAACT<br>R- GGGTTTTCCACTGCTCCAACT	
NONHSAT 231357.1	F- TGTAACTCTTTGGCAAGCCCT<br>R- TGGCCCCTGGAAGAAACTTG	F- TGGAGTTGTGAAGGACAGTGA<br>R- TGCCCTGGCTAGATTTCCAC	
NONHSAT 144427.2	F- GCCATACCTTGCAGTGTGCG<br>R- GAACGGGAGTCTGACCAGGTG	F- GCACACAGCAGGAGAGACAG<br>R- TGGGGGTCCTGAGAGTCTTC	
NONHSAT 258517.1	F- CACCCGCTTGGTAGTGTTCT<br>R- AGCTCTACCCTACACCCCAG	F- GCATTGGGCTAGTGTGAGGT<br>R- AGTGGTGGGTGTCATTTCCC	F- GGACCTCTGATAAGGCTGGC<br>R- TTTGCTCTGGCTCTTGTGGT
NONHSAT 167136.1	F- AGTGTGCGGAGTTGTGTTCA<br>R- GGGGCTCCAGGCATAAACTC		

Table S2. Primers of microarray confirmed mRNAs.

Gene	Forward primer	Reverse primer
CXCL10	TCCACGTGTTGAGATCATTGC	TCTTGATGGCCTTCGATTCTG
CXCL8	AAGAAACCACCGGAAGGAAC	AAATTTGGGGTGGAAAGGTT
FAT2	CACTCCCGAGATCCAAAGGG	GGCAGTAGAAGAGAAGCCCG
JAM2	GTCTCCTTTGTCTACTATCAAC	GGAGCCACTAATACTTCCAG
TXNIP	GCCACACTTACCTTGCCAAT	TTGGATCCAGGAACGCTAAC
ARRDC4	TGAAGAGTGGAACGAAGGCG	AGTGGGCCATTCTCCTATGC
IL-6	ACTCACCTCTTCAGAACG	GGCTTGTTCCTCACTACT
GAPDH	GTCTCCTCTGACTTCAACAGCG	ACCACCCTGTTGCTGTAGCCAA

Table S3. RT stem loop primer sequences.

microRNA	Sequence
**miR-150-3p**	GTTGGCTCTGGTGCAGGGTCCGAGGTATTCGCACCAGAGCCAACCTGTCCC
**miR-27b-5p**	GTTGGCTCTGGTGCAGGGTCCGAGGTATTCGCACCAGAGCCAACGTTCACC
**miR-297**	GTTGGCTCTGGTGCAGGGTCCGAGGTATTCGCACCAGAGCCAACCATGCAC

Table S4. Primers of miRNAs.

microRNA	Forward primer	Reverse primer
miR-150-3p	AGCTGGTACAGGCCTGG	GTGCAGGGTCCGAGGT
miR-27b-5p	GCGCAGAGCTTAGCTGATT	GTGCAGGGTCCGAGGT
miR-297	GGCGGATGTATGTGTGCAT	GTGCAGGGTCCGAGGT
U6	GCTTCGGCAGCACATATACTAAAAT	CGCTTCACGAATTTGCGTGTCAT

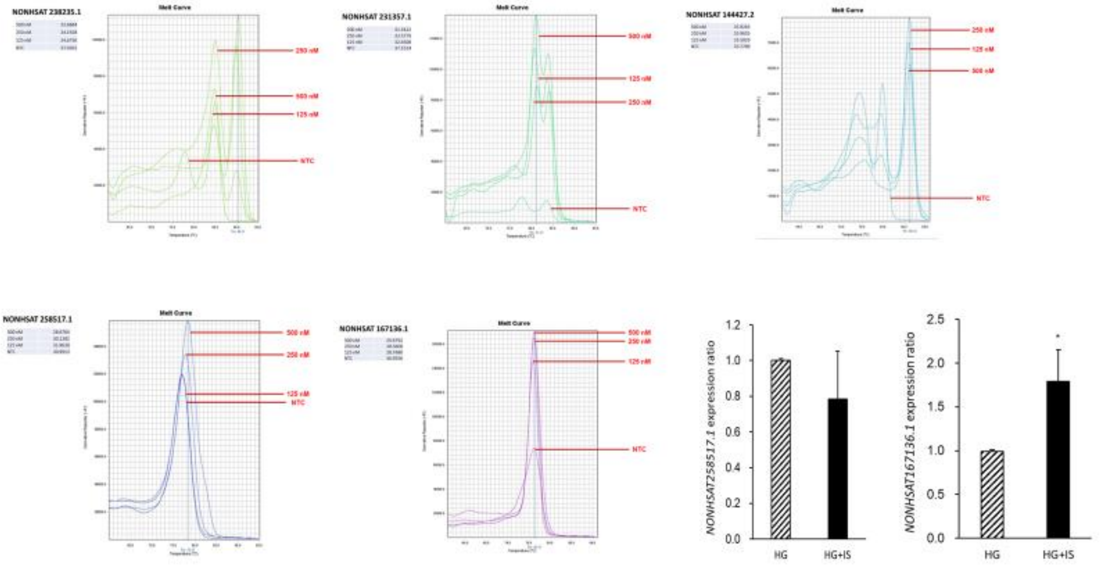

Figure S1. Primer tests of selected lncRNAs and the results of RT-PCR. To assess the designed PCR primers against the input lncRNAs sequence to determine if a product other than the desired target can be amplified, a typical denaturation (melt) curve were performed. Designed lncRNAs primers with different concentrations were evaluate that which one could be given rise to a single distinct peak in the plot of the negative derivative of fluorescence vs. temperature. The qualified designed lncRNAs primers were used to perform the RT-PCR in the condition of HUVECs explored on HG and HG+IS. LncRNAs of NONHSAT167136.1 expression was significantly ($p<0.05$) increased in HUVECs in HG+IS treatment by using the designed primer in RT-PCR experiment.

(A1) Four repeats of IP-10 western blot experiments

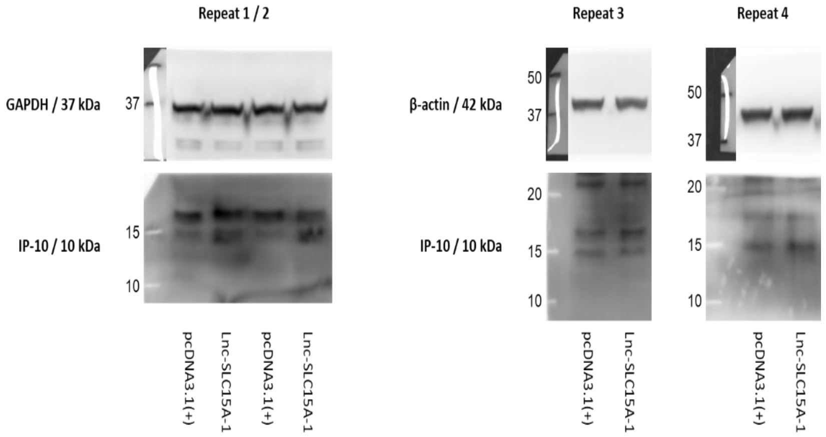

(A2) Four repeats of IL-8 western blot experiments.

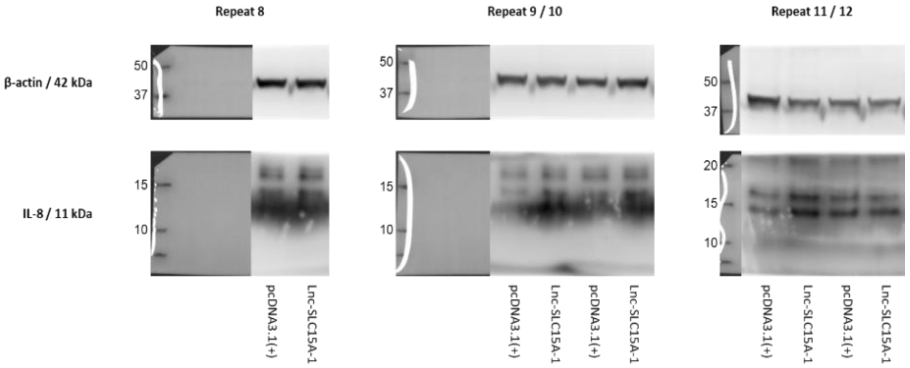

Figure S2. Original western blot gel of IP-10 and IL-8.
